# Supplementary material for: The interactome of intact mitochondria by cross-linking mass spectrometry provides evidence for coexisting respiratory supercomplexes
Source: Mol Cell Proteomics. 2017 Dec 8;17(2):216–32. doi: 10.1074/mcp.RA117.000470 (PMC5795388; doi:10.1074/mcp.RA117.000470)
Supplement: Supplemental Data [file supp_RA117.000470_133922_0_supp_23978_fzffwf.zip › spectra_annotation/mito_spectra_annotation/40-1-6-1-14-1.pdf]

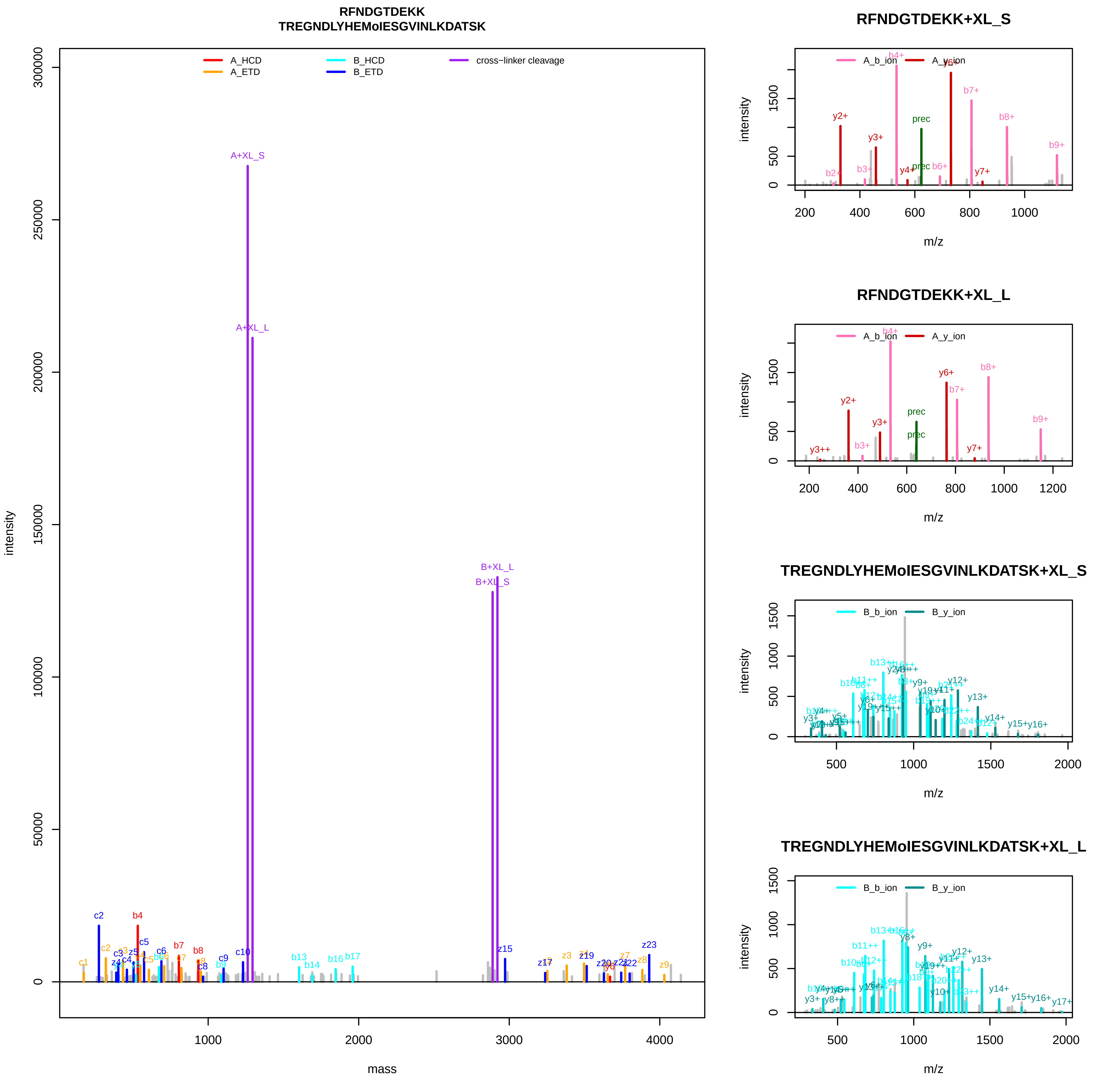
RFNDGTDEKK
TREGNDLYHEMoIESGVINLKDATSK
A_HCD
A_ETD
B_HCD
B_ETD
cross−linker cleavage
A+XL_S
A+XL_L
B+XL_L
B+XL_S
c1
c2
c3
c4
c5
c6
c8
c9
c10
b4
b7
b8
b13
b14
b16
b17
z15
z17
z19
z23
z3
z7
z8
z9
y6
intensity
mass
RFNDGTDEKK+XL_S
A_b_ion
A_y_ion
b2+
b3+
b4+
b6+
b7+
b8+
b9+
y2+
y3+
y4+
y6+
y7+
prec
m/z
RFNDGTDEKK+XL_L
y3++
b3+
y2+
y3+
b4+
prec
y6+
b7+
b8+
y7+
b9+
TREGNDLYHEMoIESGVINLKDATSK+XL_S
B_b_ion
B_y_ion
y12+
y13+
y14+
y15+
y16+
TREGNDLYHEMoIESGVINLKDATSK+XL_L
b11++
b13++
y8+
y9+
y12+
y13+
y14+
y15+
y16+
y17+
